# Role of glycogen synthase kinase-3 beta in the inflammatory response caused by bacterial pathogens

**DOI:** 10.1186/1476-9255-9-23

**Published:** 2012-06-12

**Authors:** Ricarda Cortés-Vieyra, Alejandro Bravo-Patiño, Juan J Valdez-Alarcón, Marcos Cajero Juárez, B Brett Finlay, Víctor M Baizabal-Aguirre

**Affiliations:** 1 Centro Multidisciplinario de Estudios en Biotecnología, Facultad de Medicina Veterinaria y Zootecnia, Universidad Michoacana de San Nicolás de Hidalgo, Morelia, Michoacán, Mexico; 2 Michael Smith Laboratories, The University of British Columbia, Vancouver, BC, V6T 1Z4, Canada; 3 Centro Multidisciplinario de Estudios en Biotecnología, Facultad de Medicina Veterinaria y Zootecnia, Universidad Michoacana de San Nicolás de Hidalgo, Km. 9.5 s/n Carretera Morelia-Zinapécuaro, La Palma, Tarímbaro, C.P. 58893, Morelia, Michoacán, Mexico

**Keywords:** GSK3β, NF-κB, Inflammation, Virulence factors, Bacterial infection

## Abstract

Glycogen synthase kinase 3β (GSK3β) plays a fundamental role during the inflammatory response induced by bacteria. Depending on the pathogen and its virulence factors, the type of cell and probably the context in which the interaction between host cells and bacteria takes place, GSK3β may promote or inhibit inflammation. The goal of this review is to discuss recent findings on the role of the inhibition or activation of GSK3β and its modulation of the inflammatory signaling in monocytes/macrophages and epithelial cells at the transcriptional level, mainly through the regulation of nuclear factor-kappaB (NF-κB) activity. Also included is a brief overview on the importance of GSK3 in non-inflammatory processes during bacterial infection.

## Background

Glycogen synthase kinase 3 (GSK3), in its two isoforms GSK3α and GSK3β, is a multifunctional Ser/Thr kinase found in eukaryotes [1]. This enzyme phosphorylates and regulates the function of more than 50 substrates [2] and it is a point of convergence for numerous cell-signaling pathways involved in various essential cellular functions, such as glycogen metabolism, cell cycle control, apoptosis, embryonic development, cell differentiation, cell motility, microtubule function, cell adhesion and inflammation [1-3]. The view of GSK3β has changed from an obscure metabolic kinase to an enzyme that profoundly regulates many components of the innate and adaptive immune systems. The broad array of immune actions affected by GSK3β is partly attributable to the remarkable number of crucial transcription factors that it regulates [4]. The main objective of this review is to show the importance of GSK3β in innate immunity against bacterial infections through regulation of the inflammatory response induced by virulence factors.

### General properties of GSK3

There are two major mammalian GSK3 protein isoforms (α and β) encoded by two distinct genes (*gsk3α* and *gsk3β*) [5] that are highly homologous within their kinase domains (approximately 98% of identity), but with only 36% identity in the last 76 C-terminal amino acid residues [5]. Both isoforms are structurally similar but not functionally identical because ablation of the GSK3β isoform in mice resulted in embryonic lethality via hepatocyte apoptosis. The inability of GSK3α to rescue the GSK3β-null mice indicates that the degenerative liver phenotype arises specifically from the loss of the beta isoform. Although severe hepatocyte cell death could be due to β–catenin inhibition of NF-κB, increased amount of β–catenin in GSK3β (-/-) cells was not found [6]. Physical inhibitory interaction between β–catenin and NF-κB is likely a mechanism for tumor size progression mediated by β–catenin [7]. Alternatively, GSK3α knockout mice are viable but display enhanced glucose and insulin sensitivity accompanied by reduced fat mass [8]. Mechanisms that regulate GSK3 activity are not yet fully understood. The precise control appears to be achieved by a combination of intracellular localization, phosphorylation, and interactions with GSK3 binding proteins [2]. In this regard, GSK3 has been traditionally considered a cytosolic protein; however, it is also present in the nucleus and mitochondria, where it is highly active compared with the cytosolic form [9].

**Figure 1:**
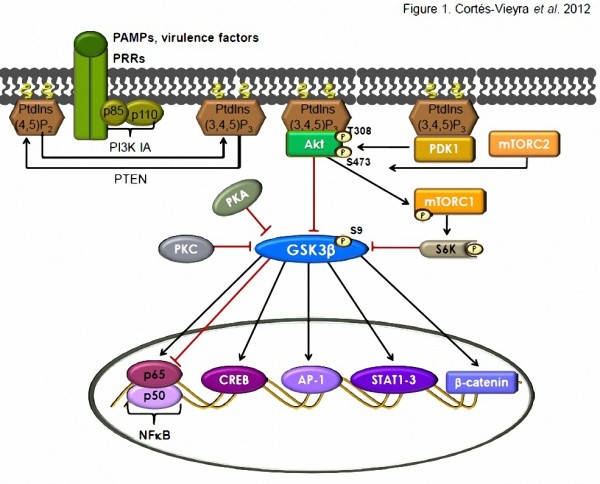
**GSK3β regulation of transcription factors activity is important for the modulation of inflammatory responses.** Pattern ecognition receptors (PRRs) activation by pathogen-associated molecular patterns (PAMPs) or virulence factors recruits class IA of phosphoinositide 3-kinases [PI3K IA (p85–p110)] to the membrane by direct interaction of the p85 subunit with the activated receptors or by interaction with adaptor proteins associated with the receptors [3,11]. The activated p110 catalytic subunit converts phosphatidylinositol-4,5-bisphosphate [PtdIns(4,5)P_2_ to phosphatidylinositol-3,4,5-trisphosphate [PtdIns(3,4,5)P_3_, providing docking sites for the signaling proteins 3’-phosphoinositide-dependent kinase 1 (PDK1) and protein kinase B (PKB/Akt) that have pleckstrin homology domains [11]. Phosphatase and tensin homologue (PTEN) antagonizes the PI3K action by dephosphorylating [PtdIns(3,4,5)P_3_[11]. Akt is phosphorylated and activated by PDK1 and the mammalian target of rapamycin complexes 2 (mTORC2) at Thr308 and Ser473, respectively, and then is able to phosphorylate and inactivates glycogen synthase kinase-3 beta [GSK3β (S9)] [11]. GSK3β can be also phosphorylated and inactivated by protein kinase A/C (PKA/C) and by the mammalian target of rapamycin complexes 1 (mTORC1) through ribosomal protein 6 kinase (S6K) [3,10]. Inactivation of GSK3β results in the activation of transcription factors such as nuclear factor-κB (NF-κB), cAMP-response element binding protein (CREB), activator protein 1 (AP-1), signal transducers and activators of transcription 1-3 (STAT1-3) and β-catenin that are involved in the regulation of the inflammatory responses [3,12,13]. GSK3β regulation on NF-κB is complex due to cell-, stimulus-, and promoter-selective interactions that might be stimulatory or inhibitory [4].

The crystal structure of GSK3β has provided insight into both the regulation of its activation and its inhibition by phosphorylation [1]. GSK3 is activated by phosphorylation of Tyr216 (GSK3β) or Tyr279 (GSK3α) and it is inactivated by phosphorylation of Ser9 (GSK3β) or Ser21 (GSK3α). Several protein kinases can phosphorylate Ser9 and Ser21, including the protein kinase B (PKB/Akt), protein kinase A (PKA), protein kinase C (PKC) and ribosomal protein 6 kinase (S6K) [2,10]. The inactivation of GSK3β by phosphorylation, carried out mainly by Akt, may result in the activation of transcription factors such as AP-1 (Jun family), cAMP-response element binding protein (CREB), signal-transducer and activator of transcription 1-3 (STAT1-3), β-catenin, and nuclear factor-kappaB (NF-κB) in response to bacterial infections [2,3] (Figure 1).

NF-κB plays a critical role in the inflammatory response and it has been traditionally used as an indicator of pro-inflammatory gene expression in cells exposed to bacterial infections. When an inflammatory stimulus induces the phosphorylation of IκB by the IκB kinase (IKK) complex, the NF-κB heterodimer (p50/p65) is free to translocate to the nucleus and activates pro-inflammatory gene expression. GSK3β is important for the modulation of NF-κB because p65 (RelA), p105 (NF-κB1) and B-cell lymphoma 3-encoded protein (BCL-3) (a transcriptional co-activator of NF-κB p50 homodimer) are phosphorylated *in vitro* by this kinase [12,14]. GSK3β promotes a rapid NF-κB activation wave by targeting the TNFα-/p65-dependent pathway and limiting NF-κB activation in BCL-3-dependent pathways [10] stabilizing and preventing p105 degradation in unstimulated cells [15]. However, GSK3β also catalyzes the phosphorylation of p105, which in turn activates the phosphorylation and degradation of IKK upon tumor necrosis factor alpha (TNF-α) treatment [15].Therefore, in basal or stimulated cells GSK3β plays a double function upon p105 [15]. Moreover, GSK3 plays distinct roles in the regulation of NF-κB, depending on the physiological state of the cell. This enzyme promotes survival and stimulates the activity of NF-κB in cells treated with TNF-α or in tumor cells in which the NF-κB pathway is constitutively active. In contrast, in quiescent cells GSK3 suppresses the expression of growth factor-inducible genes and induces apoptosis or cell cycle arrest by inhibiting both the IKK-phosphorylation of IκBα and the nuclear translocation of p50 and p65 subunits of NF-κB [16].

In view of the contrasting effects that GSK3 plays as a functional regulator of the cell activity, the following sections of this review discuss our current knowledge about the importance of GSK3β as a regulator of the inflammatory process triggered by bacterial virulence factors. Also, in the last section a brief overview on the non-inflammatory phenomena induced by bacteria is presented, which are correlated with the activity of GSK3.

### The inflammatory response

Inflammation is the body’s primary response to infection or injury and is critical for both innate and adaptive immunity. Upon infection, a variety of cytokines, chemokines, lipid mediators and bioactive amines are secreted by resident tissue cells, primarily macrophages, dendritic cells, natural killer cells, and mast cells. These factors immediately trigger a local increase of blood flow, capillary permeability and recruitment of additional circulating leukocytes via extravasation. This acute inflammatory response is characterized by the arrival of neutrophils, monocytes that differentiate into macrophages at the site of inflammation, and dendritic cells. This process is complex and involves many different signaling pathways. Most of our knowledge about pro-inflammatory signaling pathways has been obtained from studying the molecules of signaling pathways that are initiated by the activation of tumor necrosis factor receptor (TNFR), interleukin 1 receptor (IL1R), and Toll-like receptors (TLRs) [17]. Activation of TLRs by a variety of pathogen associated molecular patterns (PAMPs) or virulence factors can induce the expression of inflammatory cytokines and other molecules that help to eliminate pathogens and instruct pathogen-specific adaptive immune responses [18]. Cytokines, primarily derived from mononuclear phagocytic cells and other antigen-presenting cells (APCs), are effective in promoting the cellular infiltrate and tissue damage characteristic of inflammation. Monocytes are potently triggered to produce cytokines through the stimulation of pattern recognition receptors (PRRs). The pro-inflammatory cytokines predominantly produced by monocytes include TNF, IL-1, IL-6, CXCL8 (IL-8) and other members of the chemokine family IL-12, IL-15, IL-18, IL-23 and IL-27 [19].

During inflammation, leukocytes amplify the response but excessive or prolonged inflammation may cause damage to the host. In normal circumstances, the immune system has several mechanisms to resolve the inflammatory responses that require the termination of pro-inflammatory signaling pathways and clearance of inflammatory cells, allowing the restoration of normal tissue function. Failure of these mechanisms may lead to chronic inflammation and disease [20]. In addition to cytokines that stimulate cytotoxic, cellular, humoral, and allergic inflammation, several cytokines have predominantly anti-inflammatory effects, including IL-1Ra, TGF-β, IL-10 and IL-35 [21]. Recently, a number of reports have documented that GSK3β activity is crucial to regulate the inflammatory response either promoting or inhibiting the process through the expression of pro- or anti-inflammatory cytokines.

### Inhibition of inflammation by inhibition of the GSK3β activity

Several studies have demonstrated that inflammation is regulated by the TLR-dependent activation of PI3K-Akt signaling pathway [3,22-26]. A breakthrough paper by Martin et al. [27] established that the PI3K-Akt-dependent inhibition of GSK3β activity in human monocytes, stimulated with lipopolysaccharide (LPS), differentially affected the nature and magnitude of the inflammatory response through the activation of TLR2. This in turn resulted in the production of the anti-inflammatory cytokine IL-10, while production of pro-inflammatory cytokines IL-1β, IL-6, TNF, IL-12 and IFN-α fell substantially. Inhibition of GSK3β negatively modulated the inflammatory response because it differentially affected the nuclear activity of NF-κB (p65 subunit) and CREB through the interaction with the co-activator CREB-binding protein (CBP) [27]. In a recent study carried out in monocytes stimulated with LPS, it was established that the mammalian target of rapamycin complex 1 (mTORC1) regulates the activity of GSK3β through the activation of S6K, affecting the inflammatory response by inactivation of GSK3β. Furthermore, the inhibition of GSK3β by mTORC1 affected the association of NF-κB (p65 subunit) and CBP [10].

GSK3β activity negatively regulated the level of the anti-inflammatory cytokine IL-1Ra while concurrently increased the levels of IL1β in LPS-stimulated human monocytes. The PI3K-Akt-dependent inhibition of GSK3 increased the production of IL-1Ra due to its ability to modulate the activity of extracellular-signal-regulated kinase 1/2 (ERK1/2) [28]. These results and the fact that IL-1Ra counteracts the inflammatory properties of IL-1β [29] showed that in LPS-stimulated human monocytes the inhibition of GSK3β increases the production of anti-inflammatory cytokines and reduces the expression of pro-inflammatory cytokines, confirming the model proposed by Martin et al. [27], in which GSK3β in its active form acts as a positive regulator of inflammation.

In a study with *Mycobacterium bovis* BCG as a *Mycobacterium* model, it was demonstrated that GSK3β inhibition through the PI3K-Akt signaling increased the production of IL-10 in primary human blood monocytes (PHBM) [30]. Among the cytokines induced by BCG in PHBM, IL-10 was the key factor suppressing the production of interferon-γ (IFN-γ) in response to mycobacterial infection. Moreover, IL-10 expression induced by BCG was able to suppress the IFN-γ-dependent expression of HLA-DR, an inducible MHC class II molecule whose primary function is to present peptide antigens to the immune system. These findings suggest a significant role for GSK3β in guarding against mycobacterial evasion of host immunity, via IL-10 expression.

The PI3K-Akt signaling pathway activation following the nucleotide oligomerization domain 2 (Nod2) recognition of the agonist muramyldipeptide (MDP), a structure from peptidoglycan (PGN), negatively regulates the NF-κB pathway and interleukin (IL)-8 expression through inactivation of GSK3β. These results suggest that the PI3K-Akt-GSK3β pathway may be involved in the resolution of inflammatory responses induced by Nod2 activation [31].

Lipoteichoic acid (LTA) is a membrane-bound cell wall component of Gram-positive bacteria and is believed to be the equivalent of LPS of Gram-negative bacteria. Treatment of human gingival fibroblasts (HGFs) with LTA activated Akt which in turn inactivated GSK-3 and promoted the accumulation of β-catenin, resulting in an increase of connexin43 expression [32]. Given that the interaction of β-catenin with NF-κB leads to a decrease of the NF-κB ability to bind DNA and induce gene expression [7,33], it is likely that the accumulation of β-catenin in LTA-stimulated HGFs causes a negative regulation of the NF-κB activity and that this gives rise to a decrease of the pro-inflammatory cytokines production [32]. It is also likely that GSK-3β inactivation might be able to modulate the transcription of specific pro-inflammatory genes containing a T-cell factor/lymphoid enhancer-binding factor (TCF/LEF) binding site in their promoter. In this regard, it was recently demonstrated that β-catenin induces pro- and anti-inflammatory responses simultaneously as a result of differential gene expression carried out by Wnt/β-catenin signaling through a TCF/LEF consensus sequence and NF-κB modulation in the context of liver cancer-related inflammation [34].

Innate immunity and inflammatory responses play central roles in the pathophysiology of myocardial ischaemia/reperfusion (I/R) injury and heart failure. In this context, it was observed that PGN administration induced cardio protection in hearts of mice subjected to ischaemia, followed by reperfusion. Activation of the PI3K-Akt-GSK3β signaling pathway and reduction of the NF-κB nuclear translocation were the main factors responsible for the protection [35]. Although one may assume that reduction of NF-κB nuclear translocation decreased swelling, this waits further demonstration.

### Inhibition of inflammation by activation of GSK3β

In neonatal mouse cardiomyocytes and heart tissue culture, LPS increased the activity of GSK3β and its inhibition with chemical and genetic inhibitors enhanced LPS-induced p65 phosphorylation at the residue Ser536 and increased TNFα expression [36]. Furthermore, in line with GSK3β dephosphorylation at Ser9, Akt phosphorylation at Thr308 was reduced in LPS-treated cardiomyocytes and chemical inhibition of PI3K-Akt attenuated LPS-induced TNFα expression. These results suggest that PI3K-Akt-dependent inactivation of GSK3β plays an important function in LPS-induced TNF-α expression.

### Induction of inflammation by inhibition of GSK3β activity

The production of pro- and anti-inflammatory cytokines by activation of TLR2 and TLR4 in macrophages is dependent upon signaling events initiated by the adaptor molecules TIR-domain-containing adaptor protein (TIRAP) and myeloid differentiation primary response gene 88 (MyD88) [13]. In contrast, inactivation of GSK3β by phosphorylation at Ser9 in macrophages occurred in the absence of MyD88 [32]. In this case, GSK3β activity was a critical component of the regulatory mechanism that controlled the levels of IFNβ in TLR4-stimulated cells both *in vitro* and *in vivo*[37]. In particular, it was shown that inhibition of GSK3β activity augmented the levels of IFNβ in LPS-stimulated macrophages whereas the ectopic expression of a constitutively active GSK3β mutant caused a reduction of the IFNβ production. Interestingly, inhibition of GSK3β controlled the cellular levels of the transcription factor c-Jun that turned out to be necessary for GSK3-mediated IFNβ production. The conclusion from these results is that GSK3β acts as a critical regulatory kinase that modulated the MyD88-independent synthesis of IFNβ and of MyD88-dependent production of pro- and anti-inflammatory cytokines, demonstrating the existence of a cross-talk signaling network between these two pathways with GSK3β as a central kinase [37].

The intracellular infection of monocytes and macrophages with *Burkholderia cenocepacia*, a Gram-negative bacterium associated with exacerbated inflammation [38], caused the activation of PI3K-Akt signaling that in turn inactivated GSK3β and enhanced NF-κB activity, with the subsequent production of pro-inflammatory cytokines such as, TNFα, IL-6 and IL-8. Interestingly, NF-κB activation did not require the activation of IKK or NF-κB p65 phosphorylation, indicating that the inactivation of GSK3β was the major mechanism by which PI3K-Akt modulated the NF-κB activity without affecting *B*. *cenocepacia* uptake or survival [38].

### Induction of inflammation by activation of GSK3β

A model in which IFN-γ specifically inhibits TLR2-dependent production of IL-10 in macrophages by increasing the activity of GSK3α/β, and decreasing the expression and activity of CREB and AP-1 proteins has been established [39]. Moreover, at the same time of IL-10 suppression, IFN-γ induced the expression of TNFα. In this study GSK3 and CREB/AP-1 were key players in the signaling activated by the IFN-γ receptor and TLR2.

Microglial inflammation caused by pathogenic *S. aureus* occurred through modulation of GSK3β activity that positively regulated the NF-κB-dependent production of TNFα and nitric oxide (NO) [35]. GSK3β negatively regulated IL-10 production, and this inhibition affected the protection against heat-inactivated *S. aureus*-induced microglial inflammation [40]. These authors showed that TNFα acted upstream of NO production and that inhibition of GSK3β blocked heat-inactivated *S. aureus*-induced NF-κB p65 nuclear translocation.

In the study of the mechanisms by which GSK3β positively modulates the inflammatory response in LPS-stimulated microglia, Wang et al. (2010) [41] showed that inhibition of GSK3β activity by selective pharmacological inhibitors or its gene silencing by small interfering RNA suppressed TNFα production by blocking the NF-κB p65 transactivation activity through deacetylation of p65 at Lys310. In addition, these authors also demonstrated that inhibition of GSK3β blocked mixed lineage kinase 3 (MLK3) activity leading to a reduction of TNFα expression.

The role of GSK3β in modulating the β-catenin response in colon inflammation caused by pathogenic *Salmonella* Typhimurium was examined by using a streptomycin-pretreated mouse model [33]. *S*. Typhimurium induced an increase in β-catenin phosphorylation by augmenting GSK3β activity, reducing total β-catenin expression and compromising the physical cytoplasmic interaction between β-catenin and NF-κB. IκBα, the well-established negative regulator of NF-κB, was degraded in a similar manner as β-catenin after *Salmonella* infection. Following β-catenin and IκBα degradation, released NF-κB translocated to the nucleus and stimulated the production of the pro-inflammatory cytokines IL-6 and IL-8 [33]. The results of this study suggest a novel role for β-catenin as a negative regulator of NF-κB activity *in vivo*. Altogether, these data suggest that inhibition of GSK3β as well as β-catenin and IκBα stabilization provides important control points in the inflammatory cascade of colonic epithelial cells.

The mechanisms by which IFN-γ synergizes with LPS to induce iNOS/NO (important inductors in inflammatory cytokine production) biosynthesis in macrophages involve GSK3β-dependent inhibition of CREB activity and IL-10 expression [42]. IFN-γ co-administration with LPS was also used to study the inflammatory responses modulated by GSK3 in mouse primary glia cultures [43]. In this case, active GSK3 decreased the expression of chemokine CXCL2/MIP-2 and increased the expression of pro-inflammatory molecules CXCL1/KC, IL-12p40, CCL9/MIP-1γ, CCL2/MCP-1, P-Selectin and CCL5/RANTES. However and most prominently, active GSK3 promoted IL-6 expression due to the cooperative actions of STAT3 and GSK3 during neuro-inflammation. The production of IL-6 by glia was largely blocked by inhibiting the activity of STAT3 or GSK3β, revealing the strong dependence of IL-6 production on these signaling molecules [43]. These data reflect the cell’s ability to hyper-response to TLR-induced IFN-γ production regulated by GSK3β, resulting in a synergism of the inflammatory response.

The opposing functions of GSK3β in the inflammatory response described in the text are summarized in Table 1.

**Table 1:** GSK3β modulation of the inflammatory response caused by bacterial stimuli

**Bacterium or bacterial PAMP**	**Type of cell**	**GSK3β Inhibition: + pSer9**	**NF-κB Inhibition** ↓ **NF-kB Activation** ↑ **Not tested -**	**Pro or anti- inflammatory molecules Expressed**	**Pro or anti-inflammatory molecules Inhibited**	**Refs.**
		**GSK3β Activation: - pSer9 or + pTyr216**				
LPS	Human monocytes	+pSer9	**↓**	IL-10	IL-1β, IL-6, TNF, IL-12, IFN-α	[10,27]
LPS	Human monocytes	+pSer9	**-**	IL-1Ra	IL-1β	[28]
*Mycobacterium bovis*	Primary human monocytes	+pSer9	**-**	IL-10	IFN-γ	[30]
Muramyl dipeptide	Human embryonic kidney epithelial cells	+pSer9	**↓**	**-**	IL-8	[31]
LPS	Neonatal mouse cardiac cells		**↓**	-	TNF-α	[36]
LPS	Mice macrophages	+ pSer9	**-**	IFNβ	**-**	[37]
*Burkholderia cenocepacia*	Human monocytes and mouse macrophages	+ pSer9	**↑**	TNF-α, IL-6, IL-8	**-**	[38]
Pam_3_Cys^b^ and IFN-γ	Human macrophages	- pSer9	**-**	TNFα	IL-10	[39]
*Staphylococcus aureus*	Murine microglia	+ pTyr216	**↑**	TNF-α, NO	IL10	[40]
LPS	Murine microglia	A	**↑**	TNF-α	**-**	[41]
*Salmonella typhimurium*	Mouse colonic epithelial cells	- pSer9; + pTyr216	**↑**	TNF-α, IL-6	**-**	[33]
IFN-γ and LPS	Murine macrophages	+ pTyr216	**-**	iNOS, NO	IL-10	[42]
IFN-γ and LPS	Mouse primary microglia and astrocytes	A	**-**	IL-6, CXCL1, IL-12p40, CCL9, CCL2/MCP-1, P- Selectin,CCL5	CXCL2, MIP2	[43]

^A^ GSK3β phosphorylation at Ser9 or Tyr216 was not analyzed.

^b^ Synthetic PAMP.

### GSK3 regulation of non-inflammatory cellular processes activated by bacterial components

The *Helicobacter pylori* virulence factor VacA is one of the most important toxins that contributes to the pathogenesis and severity of gastric injury in infected humans [44]. Although it is still controversial whether cross-talk exists between the PI3K-Akt and Wnt pathways [45], the work of Nakayama et al. [46] showed that VacA induced two effects on β-catenin in gastric epithelial AZ-521 cells. The first one was the activation and nuclear accumulation of β-catenin following a short incubation with VacA, a process dependent on an active PI3K-Akt pathway and an inactive GSK3β. The second effect was that prolonged incubation with VacA resulted in inactivation of Akt and activation of GSK3β, which then down-regulated β-catenin activity. It was evident in this study that Wnt signaling, modulated by PI3K-Akt-GSK3β played a role in the pathogenesis of *H. pylori* infection, including the development of gastric cancer [46].

The lethal toxin (LeTx), produced by *Bacillus anthracis*, has been regarded as a key virulence factor in the pathogenesis of anthrax, causing immune paralysis, cell cycle arrest and cell death in host immune cells. These effects could contribute to the survival and proliferation of *B. anthracis* within the host. LeTx is a binary A:B toxin comprising protective antigen (PA) and lethal factor (LF) [47]. PA is a molecular transporter that allows receptor-mediated entry and release of LF into the cytosol. LF is a zinc metalloprotease that cleaves and inactivates the N-terminal region of the mitogen-activated protein kinase (MAPK) kinases MEKs1-4 and 6-7, resulting in the inactivation of most of their downstream signaling substrates [47]. In non-dividing cells (human peripheral blood mononuclear cells or mouse primary peritoneal macrophages) brief exposure to LeTx induced the cleavage of MEKs by LF, generating cell cycle arrest in G0-G1 phase by rapid down-regulation of cyclin D1/D2 and checkpoint kinase 1 [47]. LF also prevented TNF production in response to LPS. However, it was observed that recovery from the effects of LeTx can be facilitated by activating the PI3K-Akt-GSK3β signaling-mediated adaptive responses, indicating that modulation of this pathway can be beneficial against LeTx in cells depending on basal MEK1 activity for proliferation [47].

The inhibition of GSK3 via PI3K-Akt pathway has been identified in bacterial internalization processes in several host cells. For example, the invasion of HeLa cells by group B streptococcus (GBS) was associated with the activation of the PI3K and Akt kinases and GSK3α/β phosphoinibition [48]. One of the two type III secretion systems (TTSSs) of *Salmonella enterica* serovar Typhimurium triggers bacterial internalization through activation of PI3K-Akt [49]. Among the effectors proteins translocated by this TTSS, the GTPase modulator SopE/E2 and the phosphoinositide phosphatase SigD are known to play key roles in the process [49]. Using a reverse-phase protein array technology in HeLa, it was reported the SigD-dependent phosphorylation of Akt and its target GSK3β, demonstrating the importance of phosphoinhibition of GSK3β during host cell signaling events through bacterial infection [50]. Recently, the participation of PI3K-Akt-GSK3α/β pathway in *Staphylococcus aureus* internalization by endothelial cells was demonstrated. Although the role of the PI3K- Akt- dependent phosphorylation of GSK3α/β in the internalization of this bacterium was not determined in this study, phosphorylation of GSK3β at Ser9 and GSK3α at Ser21 was clearly associated with the invasion of *S. aureus* to the endothelial cells [51]. It is likely that in the internalization of GBS and *Salmonella enterica* by HeLa cells and *S. aureus* by endothelial cells, GSK3 functions by regulating the cytoskeletal rearrangement, as it was observed in macrophages RAW264.7 in which phosphorylation of paxillin at Ser126 and 130 was mediated by an ERK/GSK3 dual-kinase mechanism [52].

## Conclusions

The experimental evidence accumulated so far indicates that GSK3β plays an essential role in the regulation of the inflammatory response during the interaction between pathogenic bacteria and animal cells. The opposing effects of GSK3β on the inflammation is dependent upon the bacterium or virulence factor (*Mycobacterium bovis*, *Burkholderia cenocepacia, Staphylococcus aureus, Salmonella typhimurium,* LPS, MDP), the type of cell (epithelial cells, monocytes, cardiomyocytes, macrophages, microglia or astrocytes and fibroblasts) and probably on the physiological state of the cell [16]. Although activated NF-κB induces an inflammatory response, the active or inactive state of GSK3β modulates the activity of NF-κB, either promoting or inhibiting an inflammatory response.

Apart from its fundamental regulatory role on the inflammatory response, GSK3 is associated with bacterial internalization [48,50,51] and other processes related to the pathogenesis of the infection [46,47]. However, more studies are needed to clarify the details about the mechanisms that GSK3 employs to control the bacterial internalization, the pathogenesis of infection and the expression of genes with pro- or anti-inflammatory function.

## Abbreviations

AP-1: Activator protein 1; APCs: Antigen-presenting cells; BCL3: B-cell lymphoma 3-encoded protein; CREB: cAMP-response element binding protein; ERK1/2: Extracellular-signal-regulated kinase 1/2; GSK3α/β: Glycogen synthase kinase-3 alpha/beta; HLA-DR: Major histocompatibility complex, MHC class II, cell surface receptor; LTA: Lipoteichoic acid; IFN-γ/β: Interferon-γ/β; IL1R: Interleukin 1 receptor; IKK: IκB kinase; MAPK: Mitogen-activated protein kinase; MLK3: Mixed lineage kinase 3; mTORC1/2: The mammalian target of rapamycin complexes 1/2; MDP: muramyldipeptide; MyD88: Myeloid differentiation primary response gene 88; NF-κB: Nuclear factor-κB; NO: Nitric oxide; iNOS: inducible Nitric oxide synthase; Nod2: Nucleotide oligomerization domain 2; PAMPs: Pathogen associated molecular patterns; PDK1: 3’-phosphoinositide-dependent kinase 1; PKA/C: Protein kinase A/C; PKB: Protein kinase B; PI3K IA: Class IA of phosphoinositide 3-kinases; PTEN: Phosphatase and tensin homologue; PRRs: Pattern recognition receptors; S6K: Ribosomal protein 6 kinase; STAT1-3: Signal-transducer and activator of transcription 1-3; TCF/LEF: T-cell factor/lymphoid enhancer-binding factor; TIR: Toll/Interleukin-1 receptor; TIRAP: TIR-domain-containing adaptor protein; TLRs: Toll-like receptors; TRIF: TIR-domain-containing adapter-inducing interferon-β; TNFα: Tumor necrosis factor alpha; TNFR: Tumor necrosis factor receptor.

## Competing interests

The authors declare that they have no competing interests.

## Authors’ contributions

RCV and VMBA conceived of the review, designed, and wrote the manuscript. ABP, JJVA, MCJ and BBF contributed to critical reading and comments of the manuscript. All authors read and approved of the final manuscript.
